# Management of advanced sporadic endolymphatic sac tumours: a single-centre case series

**DOI:** 10.1007/s11845-026-04448-2

**Published:** 2026-05-18

**Authors:** N. Rajendram, N. A. O’Keeffe, J. Garry, H. Barrett, S. Looby, R. McConn Walsh

**Affiliations:** 1 Department of Otorhinolaryngology/Head & Neck Surgery, National Skull Base Centre, Beaumont Hospital, Dublin, Ireland; 2 Department of Histopathology, Beaumont Hospital, Dublin, Ireland; 3 Department of Radiology, Beaumont Hospital, Dublin, Ireland

**Keywords:** Endolymphatic sac tumour, Facial nerve, Multidisciplinary, Skull base surgery, Temporal bone tumour

## Abstract

**Background:**

Endolymphatic sac tumours (ELSTs) are rare, locally aggressive neoplasms of the temporal bone that often present at an advanced stage due to their indolent clinical course. Complete surgical excision may be technically challenging when tumours involve the jugular foramen, cerebellopontine angle (CPA) or adjacent cranial nerves and may therefore be associated with significant neurological morbidity.

**Objective:**

To evaluate facial nerve and radiological outcomes following planned subtotal resection with selective use of adjuvant radiotherapy in patients presenting with advanced sporadic ELST.

**Methods:**

A retrospective review was performed of patients diagnosed with ELST and managed at the National Skull Base Centre, Beaumont Hospital. Clinical presentation, radiological findings, surgical approach, extent of resection, histopathological features, adjuvant therapy and postoperative outcomes were analysed. All patients underwent screening for von Hippel–Lindau (VHL) disease. Facial nerve function was assessed using the House-Brackmann (HB) grading system. Radiological stability was evaluated using serial postoperative magnetic resonance imaging (MRI).

**Results:**

Two patients presented with locally advanced ELST involving the petrous temporal bone and skull base with cranial nerve involvement at diagnosis. Both patients underwent screening for von Hippel-Lindau disease. Both underwent transmastoid subtotal resection to preserve neurological function. One patient subsequently received adjuvant radiotherapy, while the second was managed with radiological surveillance alone. Patient 2 improved from House–Brackmann grade V to grade II, while Patient 1 maintained grade II function. Serial postoperative MRI demonstrated stable residual disease.

**Conclusion:**

In selected cases of advanced sporadic ELST, planned subtotal resection with selective adjuvant radiotherapy may preserve cranial nerve function while maintaining short-term radiological stability.

## Introduction

Endolymphatic sac tumours (ELSTs) are rare, locally aggressive neoplasms arising from the endolymphatic sac of the posterior petrous temporal bone and may present at an advanced stage due to their slow growth and non-specific clinical symptoms [1, 2]. Patients frequently present with skull base extension or cranial nerve involvement at the time of diagnosis [3]. ELSTs may occur sporadically or in association with von Hippel-Lindau (VHL) disease. Immunohistochemical profiling has improved diagnostic accuracy in distinguishing ELST from other papillary tumours of the temporal bone [4].

Gross total resection remains the standard treatment; however, tumours involving the jugular foramen, cerebellopontine angle (CPA) or adjacent neurovascular structures may pose significant operative challenges [5].

A multidisciplinary approach incorporating maximal safe resection with selective use of adjuvant radiotherapy may therefore be considered in patients presenting with advanced disease [6].

## Methods

A retrospective review was performed of patients diagnosed with ELST and managed at the National Skull Base Centre, Beaumont Hospital, Dublin. Patients presenting with radiologically and histologically confirmed ELST were identified through institutional records.

Cases were identified through multidisciplinary skull base meeting records and histopathology databases between 2000 and 2025.

Demographic data, clinical presentation, radiological findings, surgical approach, extent of resection, histopathological characteristics, adjuvant therapy and postoperative outcomes were reviewed. All patients underwent screening for von Hippel–Lindau (VHL) disease.

All cases were discussed at a multidisciplinary skull base meeting comprising otolaryngology, neurosurgery, radiology, oncology, and histopathology specialists to determine optimal management strategy. Surgical approach was selected based on tumour extent and cranial nerve involvement with the intention of planned maximal safe resection while preserving neurological function.

Facial nerve function was assessed pre- and post-operatively using the House-Brackmann grading system. Radiological outcomes were evaluated using serial postoperative MRI, with stability defined as the absence of interval tumour progression. Patients underwent postoperative MRI at 6 months, followed by serial imaging at 6–12 month intervals thereafter. Follow-up remains ongoing in both patients, with serial postoperative MRI demonstrating stable residual disease to date.

This study was conducted as a retrospective review of anonymised patient data in accordance with institutional clinical audit guidelines.

Written informed consent for publication of clinical data and images was obtained from both patients. Ethical approval was obtained from the Beaumont Hospital Research Ethics Committee.

## Results

Two patients presented with locally advanced endolymphatic sac tumours involving the petrous temporal bone and skull base, with cranial nerve involvement at diagnosis. Both patients underwent screening for von Hippel-Lindau disease with negative results.

Both patients were male (aged 55 and 49 years) presenting with advanced ELST involving the petrous temporal bone with jugular foramen and CPA extension. Immunohistochemistry demonstrated CK7, CD56 and PAX8 positivity in both cases. Follow-up duration was 6 years and 18 months respectively, with stable disease on serial imaging.

## Clinical presentation

### Patient 1

**Fig. 1:**
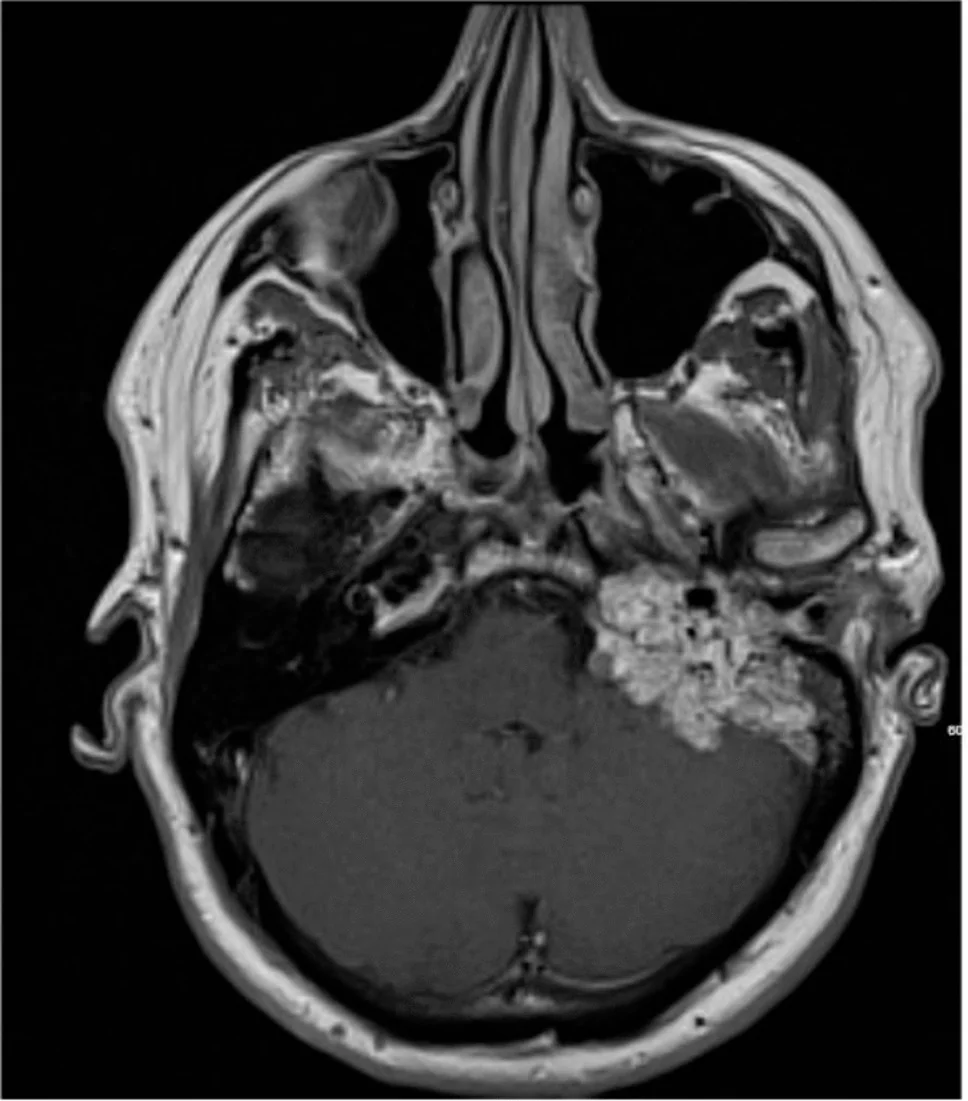
Axial T1-weighted post-gadolinium magnetic resonance imaging obtained prior to treatment, demonstrating a heterogeneously enhancing mass centred in the left posterior petrous temporal bone in the region of the endolymphatic sac, with extension into the cerebellopontine angle and toward the jugular foramen

**Fig. 2:**
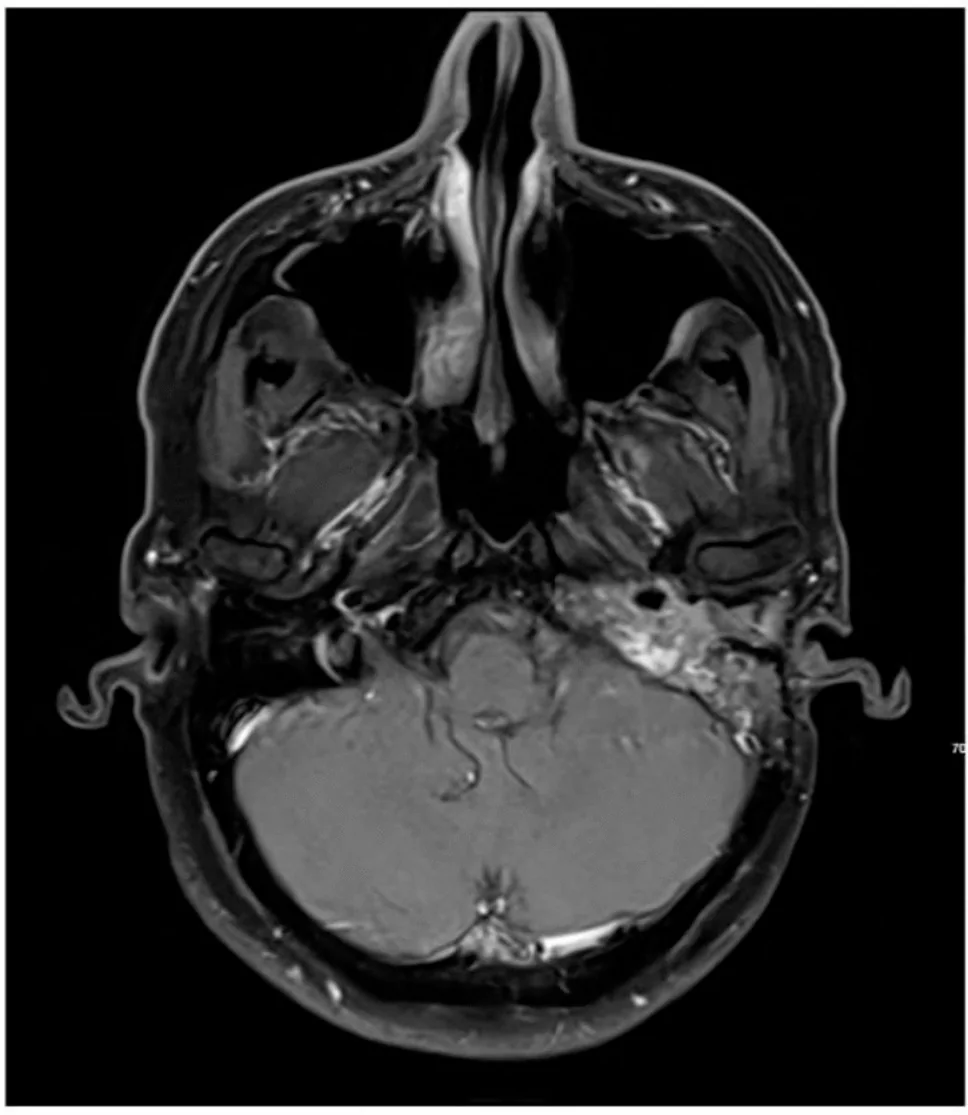
Axial T1-weighted post-gadolinium MRI obtained following surgical debulking and adjuvant radiotherapy, demonstrating decreased size of the extraosseous enhancing component of the left petrous temporal bone lesion

A 55-year-old male presented with a one-month history of left-sided otalgia and cranial nerve palsies involving VII, IX and X. Otoscopic examination revealed a vascular mass occupying the external auditory canal. Pure tone audiometry demonstrated left-sided severe-to-profound sensorineural hearing loss. CT and MRI demonstrated an extensive destructive lesion centred on the left petrous temporal bone with extension to the internal auditory meatus and jugular foramen with erosion of the ossicular chain and tegmen tympani. MRI findings are illustrated in Figs. 1 and 2.

### Patient 2

**Fig. 3:**
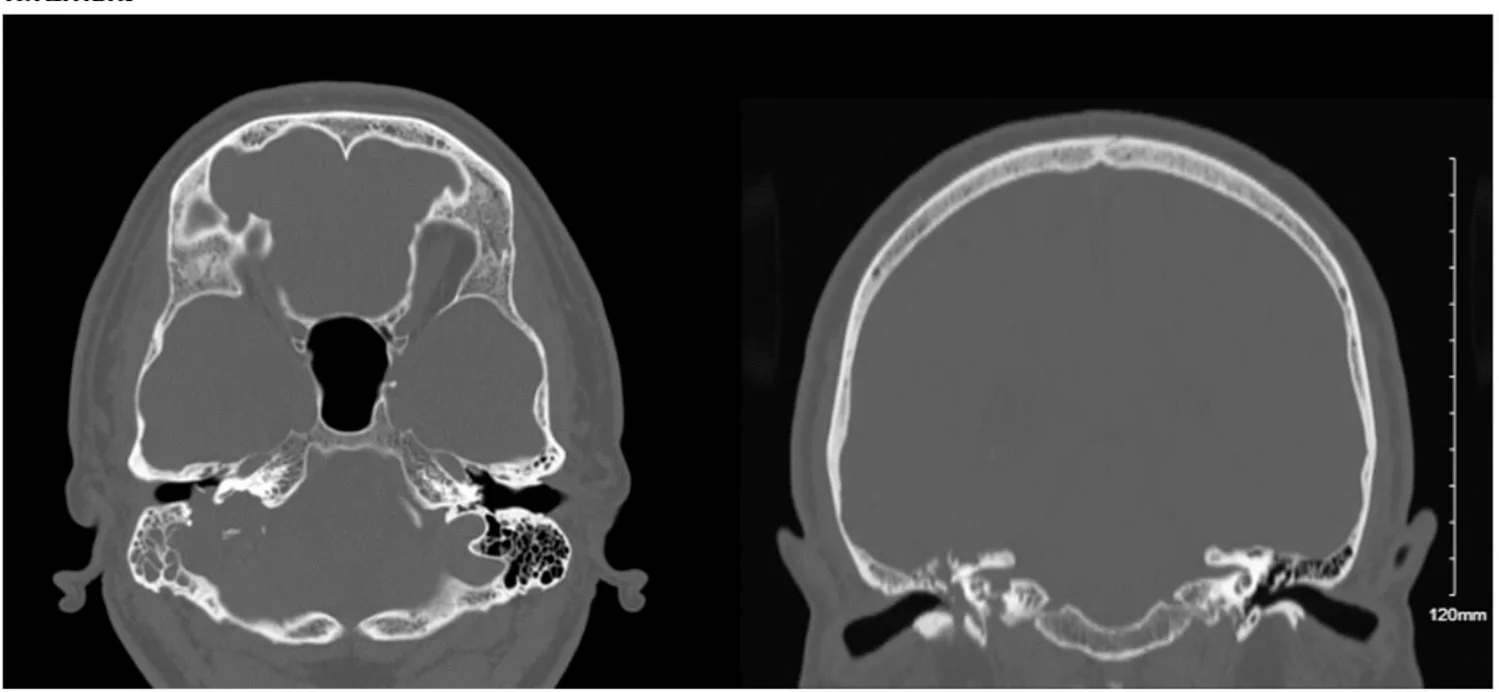
Axial and coronal CT of the temporal bone demonstrating a large locally invasive right cerebellopontine angle extra-axial mass with destruction of the posterior petrous temporal bone. The lesion is inseparable from the sigmoid sinus and demonstrates extensive osseous erosion, with involvement of the facial nerve canal

**Fig. 4:**
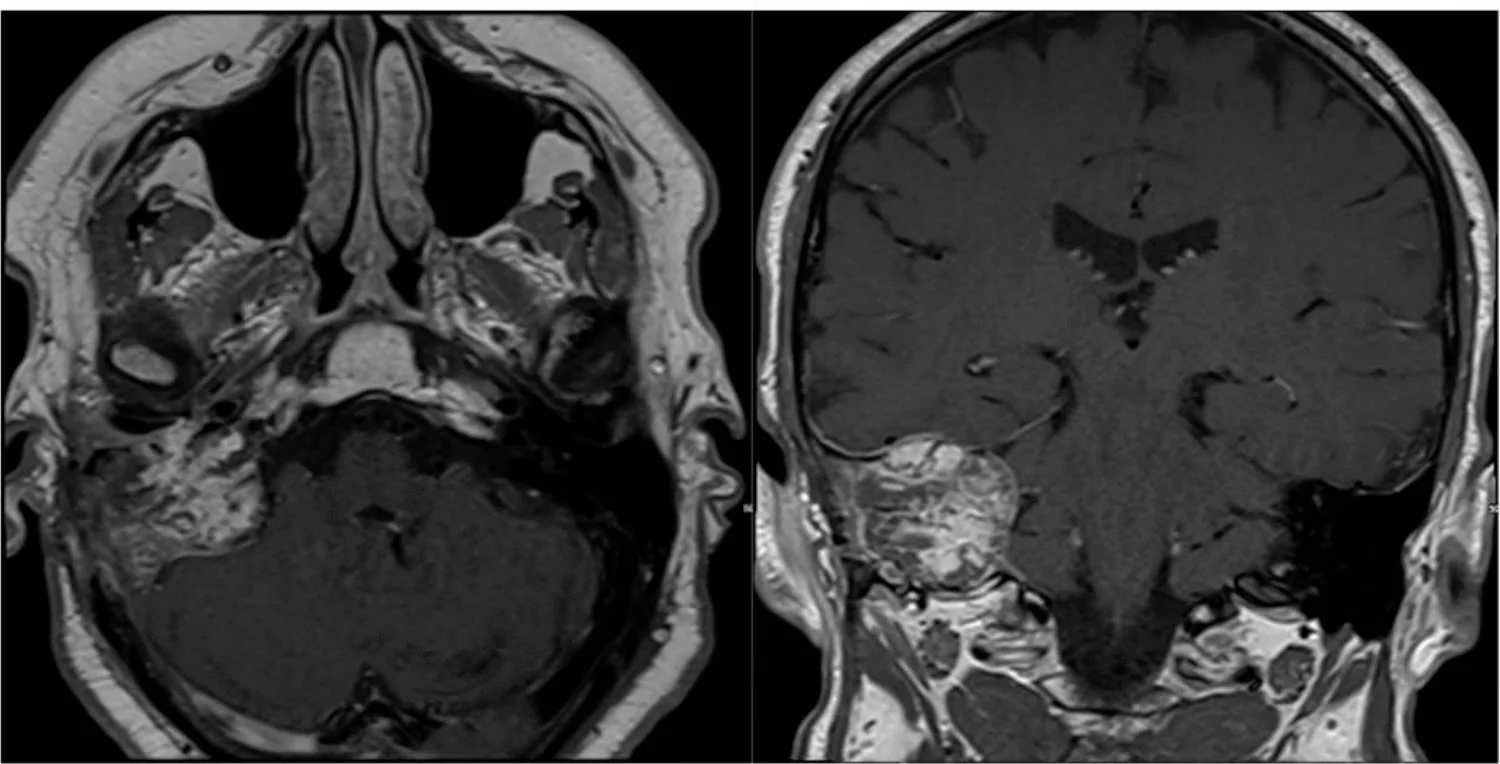
Axial and coronal post-contrast T1-weighted MRI demonstrated a heterogeneously enhancing lesion centred on the right endolymphatic sac with extension into the cerebellopontine angle, middle ear cavity, inner ear and internal auditory canal. The lesion measured 3 × 3 × 2.8 cm, demonstrated multiple cystic components with areas of diffusion restriction and showed no interval increase in size compared with prior imaging

A 49-year-old male presented with progressive right facial weakness and hearing loss over four months. Clinical examination demonstrated a House–Brackmann (HB) grade V facial nerve palsy and a vascular mass arising from the posterior external auditory canal. Pure tone audiometry demonstrated a severe-to-profound sensorineural hearing loss. CT and MRI demonstrated an extensive destructive lesion centred within the right petrous temporal bone with extension into the cerebellopontine angle and jugular foramen (Figs. 3 and 4).

### Operative management and outcomes

**Fig. 5:**
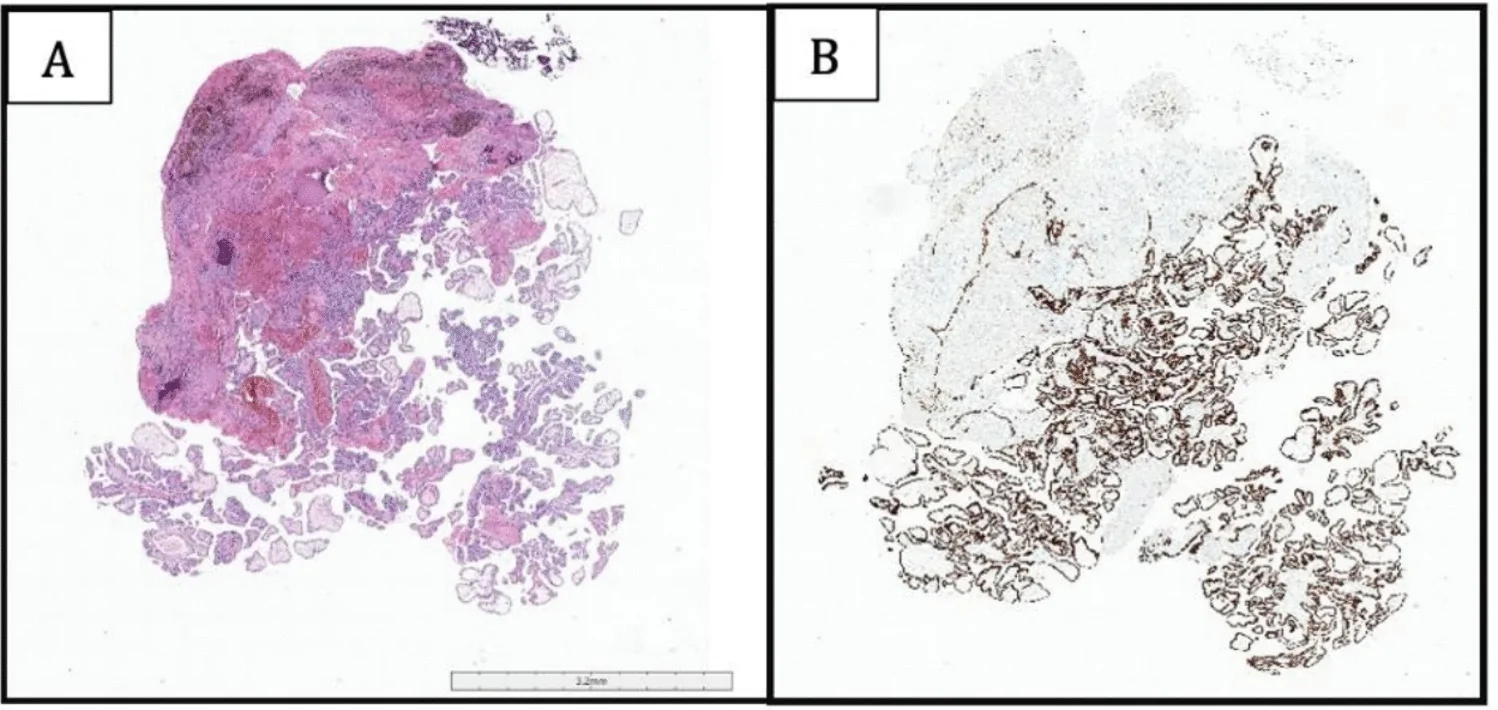
**A** Haematoxylin and eosin staining demonstrating papillary architecture with fibrovascular cores lined by bland epithelial cells. **B** Immunohistochemical staining demonstrating positivity for CK7 supporting the diagnosis of endolymphatic sac tumour

Both patients underwent transmastoid subtotal tumour resection with the intention of preserving neurological function. Patient 1 subsequently underwent adjuvant fractionated external beam radiotherapy to the residual tumour bed following multidisciplinary discussion. Patient 2 opted for conservative management with no radiation therapy. Histopathological examination confirmed endolymphatic sac tumour with characteristic papillary architecture and positive immunostaining for CK7 and PAX8 (Fig. 5).

Postoperatively, Patient 2 demonstrated improvement in facial nerve function from House–Brackmann grade V to grade II, while Patient 1 maintained House–Brackmann grade II function. Serial postoperative MRI surveillance has demonstrated stable residual disease in both patients to date.

## Discussion

Endolymphatic sac tumours represent a rare and diagnostically challenging pathology of the petrous temporal bone, often presenting at an advanced stage with cranial nerve involvement [1]. They rarely metastasise and therefore primarily represent a problem of local disease control [7]. Patients frequently present late due to relatively non-specific early symptoms [8]. The estimated incidence in the general (adult) population is approximately 1 in 30,000 individuals, although precise population based data are lacking [9]. ELSTs are best described in von Hippel–Lindau cohorts, where prevalence ranges from 3 to 11% [10]; however, sporadic ELSTs are exceedingly rare, with the literature largely limited to isolated case reports and small case series, in contrast to the better-characterised VHL-associated cohort. This highlights the importance of reporting institutional experience to inform management strategies.

Tang et al. reported favourable local control following gross total resection, with recurrence more commonly observed in cases of residual disease; however, facial nerve morbidity was significant in advanced cases [8]. In contrast, subtotal resection combined with adjuvant radiotherapy has demonstrated acceptable tumour stability with improved functional preservation in selected cohorts [11]. This approach may be particularly relevant in patients presenting with advanced tumours involving the jugular foramen or cerebellopontine angle where aggressive attempts at gross total resection risk permanent cranial nerve deficits; therefore, a strategy of planned subtotal resection with surveillance or adjuvant radiotherapy may represent a pragmatic balance between oncologic control and functional preservation.

While complete surgical excision has traditionally been considered the mainstay of treatment, gross total resection may be difficult to achieve in tumours with extension to the jugular foramen, cerebellopontine angle (CPA) or adjacent cranial nerves without incurring significant neurological morbidity, particularly involving the facial and lower cranial nerves [5, 6]. Postoperative radiotherapy is frequently used as adjuvant therapy following subtotal resection and may also serve as salvage treatment in recurrent disease [12, 13].

The histological differential diagnosis of ELST includes choroid plexus papillomas, paragangliomas, papillary ependymoma, middle ear adenoma and metastatic carcinoma from kidney, prostate or thyroid. Diagnostic distinction can be challenging due to overlapping papillary architecture and occasional clear cell or vascular features. Metastatic renal cell carcinoma (RCC) is particularly important to exclude, as it may closely resemble ELST morphologically and immunohistochemically and must be ruled out clinically and radiologically. Paragangliomas show neuroendocrine marker positivity (synaptophysin, chromogranin), which is absent in ELST. Papillary ependymomas demonstrate pseudorosettes and are GFAP-positive. Choroid plexus papillomas arise within the ventricular system, do not invade bone, and demonstrate transthyretin positivity. Metastatic papillary and follicular thyroid carcinomas express TTF-1 and thyroglobulin, while metastatic prostate carcinoma is PSA-positive, allowing immunohistochemical differentiation [3, 4, 14].

On CT imaging, endolymphatic sac tumours typically demonstrate bony erosion centred on the posteromedial petrous temporal bone often with a thin calcified peripheral rim reflecting expanded cortex; smaller lesions may show only subtle abnormalities such as vestibular aqueduct widening. CT also helps differentiate ELST from jugulotympanic lesions by demonstrating preservation of the jugular foramen architecture and characteristic sparing of the cochlea and middle ear [12, 14]. On MRI, ELSTs are usually heterogeneous, with multiple high-signal foci on T1- and T2-weighted sequences corresponding to blood products, methaemoglobin, proteinaceous cysts, or cholesterol clefts, features that are uncommon in other temporal bone tumours. Low-signal foci may reflect hemosiderin deposition from chronic haemorrhage, while calcifications and vessels produce signal voids. Post-contrast imaging typically shows heterogeneous enhancement of the solid tumour components [13, 15, 16].

Both patients in our series presented with advanced sporadic ELST involving the petrous temporal bone and skull base, with radiological evidence of CPA and jugular foramen extension. In this context, the primary surgical objective was maximal safe resection while preserving cranial nerve function, rather than radical excision. Subtotal tumour resection was therefore performed in both cases with the intention of maintaining facial nerve integrity, followed by adjuvant radiotherapy as part of a multidisciplinary management strategy.

ELSTs are considered relatively radiosensitive and adjuvant radiotherapy has been employed following subtotal resection or in unresectable disease to improve local tumour control. In this context, planned subtotal resection followed by adjuvant radiotherapy may represent a pragmatic management strategy in advanced sporadic ELST where gross total resection would carry an unacceptable risk of neurological morbidity [12].

This approach was associated with preservation or improvement of postoperative facial nerve function, with one patient demonstrating improvement from House-Brackmann grade V to grade II. Serial postoperative MRI surveillance has demonstrated stable residual disease in both patients to date.

In patients presenting with late-stage sporadic ELST a multidisciplinary skull base approach incorporating subtotal resection and postoperative radiotherapy may represent a pragmatic strategy to balance tumour control with preservation of neurological function.

Ongoing radiological surveillance remains essential given the potential for delayed tumour progression. Given the rarity of sporadic ELST, small institutional case series remain valuable in refining management strategies and informing multidisciplinary decision-making.

This study is limited by the small sample size and ongoing follow-up, which precludes conclusions regarding long-term tumour control. Nevertheless, this series highlights a pragmatic multidisciplinary strategy for advanced sporadic ELST, as radical resection in such cases may carry an increased risk of treatment-related neurological morbidity, particularly involving the facial and lower cranial nerves, thereby reinforcing the importance of functional preservation in selected cases.

## Conclusion

ELSTs may present at an advanced stage with skull base and cranial nerve involvement, making complete surgical excision challenging without significant morbidity. In selected cases a multidisciplinary approach incorporating maximal safe resection with selective use of adjuvant radiotherapy may allow preservation of neurological function while maintaining radiological stability. Ongoing surveillance remains essential in view of the potential for delayed disease progression.
